# High-linear energy transfer radiation disrupts natural killer cell surveillance of senescent intestinal cells in the mouse intestine

**DOI:** 10.1186/s43556-026-00446-z

**Published:** 2026-04-07

**Authors:** Santosh Kumar, Shubhankar Suman, Heng-Hong Li, Jerry Angdisen, Kamal Datta, Albert J. Fornace

**Affiliations:** 1 Department of Oncology, Lombardi Comprehensive Cancer Center, Georgetown University Medical Center, Research Building, Room E504, 3970 Reservoir Rd., NW, Washington, DC 20057 USA; 2 Department of Biochemistry and Molecular & Cellular Biology, Georgetown University Medical Center, Washington, DC 20057 USA

**Keywords:** NK cells, High-LET radiation, Senescence, P38 MAPK, Qa-1b, Lamina propria

## Abstract

High-Linear Energy Transfer (LET) ion radiation, such as ^28^Si ions, is densely ionizing and poses a significant risk to astronauts during long-duration space missions. We previously showed that mice exposed to high-LET ionizing radiation (IR) exhibit greater accumulation of senescent cells in the intestine than those exposed to equivalent doses of low-LET γ-rays. However, the mechanisms driving this persistent senescence remain unclear. Given the role of Natural killer (NK) cells in senescent cell clearance, we investigated the impact of IR on intestinal NK cell function. At 60 days post-irradiation, intestinal tissues from ^28^Si-exposed mice showed a significant reduction in NKp46⁺ NK cells and decreased expression of molecules associated with NK activation and epithelial interactions. NK cell subtype analysis further revealed a decline in functionally mature populations involved in recognizing stressed cells. In parallel, intestinal epithelial cells (IECs) displayed altered expression of NK cell regulatory ligands, including reduced activating signals and increased inhibitory signaling associated with Qa-1b (non-classical MHC class Ib). Mechanistically, these changes were linked to activation of p38 Mitogen-Activated Protein Kinase (MAPK) signaling. Using irradiated intestinal organoids, we observed that pharmacological inhibition of the p38 MAPK pathway decreased Qa-1b expression and enhanced NK cell cytotoxic activity. Causality experiments further demonstrated that Qa-1b directly regulates NK cell–mediated cytotoxicity against senescent IECs. Collectively, these findings indicate that high-LET IR compromises intestinal immune surveillance by impairing NK cell function through a p38 MAPK–Qa-1b signaling axis, providing mechanistic insight into radiation-induced immune dysregulation.

**Supplementary Information:**

The online version contains supplementary material available at 10.1186/s43556-026-00446-z.

## Introduction

The gastrointestinal tract is a highly dynamic and complex organ that serves as a vital interface between the host and its environment, playing essential roles in digestion, nutrient absorption, and immune defense [1]. Its rapidly renewing epithelial cells and dense immune cell populations make it particularly vulnerable to external stressors, including ionizing radiation (IR) [2]. While the effects of low-linear energy transfer (LET) IR, such as γ-rays, on gut pathophysiology are relatively well understood, the unique and severe biological consequences of high-LET IR, such as that encountered during space travel or ion-beam radiotherapy, remain less explored. Even at non-lethal doses, high-LET IR induces dense ionization and complex DNA damage, often leading to persistent tissue injury marked by chronic accumulation of senescent cells, similar to accelerated aging phenotype [3–9]. Senescent cells, which undergo irreversible growth arrest and secrete pro-inflammatory factors known as the Senescence-Associated Secretory Phenotype (SASP), accumulate over time and in response to IR, contributing to chronic inflammation, tissue dysfunction, and potentially tumorigenesis [6, 10, 11]. Importantly, exposure to high-LET IR resulted in an approximately 8- to 15-fold increase in senescent cell accumulation compared to an equivalent dose of low-LET γ-ray radiation, suggesting impaired clearance of senescent cells [5, 6].

Senescent cells are typically removed by immune effector cells, particularly Natural killer (NK) cells and macrophages, which possess specialized receptors and cytotoxic mechanisms that enable the recognition and elimination of stress-induced, potentially harmful senescent cells [12–14]. NK cells express activating receptors such as NK Group 2D (NKG2D) and NK cell p46-related protein (NKp46), which recognize stress-induced ligands including Retinoic Acid Early inducible gene 1 (Rae-1) and Histocompatibility 60 (H60) on target cells [15]. These ligands are typically upregulated in target cells, such as senescent intestinal epithelial cells (IECs) in response to cellular stress, promoting NK cell-mediated cytotoxicity [15]. In contrast, inhibitory receptors like NK Group 2 member A (NKG2A) and members of the Lymphocyte antigen 49 (Ly49) family bind to ligands such as Qa-1b, the murine homolog of the non-classical MHC class I molecule HLA-E, which binds NKG2A on NK cells to deliver inhibitory signals and thereby acts as a critical regulator of NK cell-mediated immune surveillance [16–19]. Additionally, NK cells employ death-inducing ligands such as TNF-related apoptosis-inducing ligand (TRAIL) and Fas ligand (FasL) to eliminate target cells [20], and rely on co-stimulatory molecules like CD27 and CD226 to amplify activation signals [21].

While high doses of low-LET IR are known to induce lymphopenia, lower, non-lethal doses result in long-term effects on NK cells, including persistent disruption of immune homeostasis. This may contribute to a chronic pro-tumorigenic and immunosuppressive microenvironment by promoting the sustained production of inhibitory cytokines and enhancing the shedding of soluble NKG2D ligands from target cells [22–24]. The role of NK cells is essential for eliminating stress-induced senescent cells to preserve tissue integrity; however, the efficacy of this clearance is frequently compromised by the high degree of senescence heterogeneity, which dynamically governed by the nature of initiating stressor and the surrounding tissue microenvironment [12–14]. Given its higher relative biological effectiveness (RBE), high-LET radiation induces more severe and persistent intestinal injury than equivalent doses of low-LET radiation. This process likely reflects distinct senescence programs driven by complex DNA damage and altered microenvironmental signaling, potentially exacerbated by impaired immune cell–mediated clearance. Based on this rationale, we hypothesized that high-LET irradiation compromises intestinal immune surveillance by altering NK cell populations and modulating the expression of key immunoregulatory ligands in IECs.

To test this hypothesis, we used a total-body irradiated mouse model together with an intestinal organoid system to investigate whether impaired immune surveillance contributes to the persistent accumulation of senescent cells in the murine intestinal mucosa following high-LET radiation exposure. We specifically examined whether high-LET irradiation disrupts activating receptor-ligand interactions or enhances inhibitory signaling pathways that suppress NK cell cytotoxicity, and we explored intrinsic stress-response pathways in IECs that may influence immune crosstalk. Mechanistically, we identify p38 Mitogen-Activated Protein Kinase (MAPK)-Qa-1b signaling axis as a critical pathway suppressing NK cell cytotoxicity, which was reversible through pharmacological inhibition in irradiated intestinal organoids. Collectively, these findings reveal a novel p38 MAPK-Qa-1b signaling axis contributing to IR-induced immune dysfunction and suggest a promising target for therapeutic intervention to restore mucosal immune surveillance and mitigate gastrointestinal pathologies associated with IR exposure.

## Results

### High-LET irradiation reduces NK cell infiltration in the lamina propria (LP) in a tissue-specific manner

**Fig. 1:**
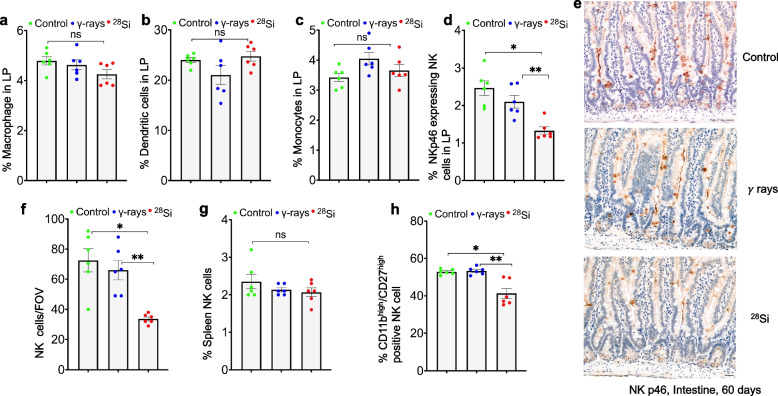
High-LET radiation alters NK cell infiltration and retention in the LP of the mouse intestine 60 days after exposure. Bar graphs showing the percentages of immune cell types among total CD45⁺ cells in the LP (*n* = 6 per group): **a** macrophages; **b** dendritic cells; **c** monocytes; and **d** NK cells. One-way ANOVA followed by Tukey’s post hoc test was performed. (*p = 0.0004; **p = 0.0096, error bar = ± SEM, n = 6). **e** Representative bright-field images of mouse intestine showing NK-cell retention in the LP. Nuclei were counterstained with hematoxylin. Scale bar, 20 μm. **f** Quantification of NK cells analyzed by one-way ANOVA with Tukey’s post hoc test. (*p = 0.0008; **p = 0.0037, error bar = ± SEM. n = 6). **g** Bar graph showing the percentages of NK cells among total CD45⁺ cells in the mouse spleen after irradiation. **h** Bar graph showing the percentage of CD11b^high^/CD27^high^ NK-cell subset among total NK cells after irradiation. Data are presented as mean ± SEM. Statistical significance: *p = 0.0007; **p = 0.0004; *n* = 6; ns = non-significant

We and others have reported that high-LET IR induces persistent oxidative stress and senescence in the mouse intestine, leading to inflammation, increased gut permeability and fibrosis, which may adversely affect immune-cell presence and function in the LP [4–6]. To analyze the effect of irradiation on immune-cell retention in the LP, isolated immune cells from the LP were stained with multiple phenotypic markers (F4/80, CD11c, CD64, NKp46) and analyzed by flow cytometry using a gating strategy described (Fig. S1a-g). A slight decrease in the macrophage population was observed following high-LET IR, while the monocyte and dendritic cell populations remained unchanged (Fig. 1a-c). In contrast, the NK cell population in the LP exhibited a radiation quality-dependent decrease after irradiation (Fig. 1d). To confirm these findings, intestinal tissue sections were immunostained for the NK cell marker NKp46. The results demonstrated a radiation quality-dependent reduction in NK cell retention in the LP compared to controls (Fig. 1e). Notably, the decrease in NK cell population was statistically significant in the high-LET group compared to both the γ-irradiated and control groups (Fig. 1f). Further, to determine whether the reduction in NK cell number was due to a systemic decline in NK cells, we analyzed NK cell populations in splenocytes that remained largely unchanged following high-LET irradiation, suggesting that the reduction in NK cell retention is specific to the LP region (Fig. 1g).

We further examined NK cell retention at an early time point (5 days) and at 60 days after irradiation. The data show a significant reduction in LP-resident NK cells at both time points. Although NK cell numbers at 5 days were lower than at 60 days, the difference was not statistically significant (Fig. S2a, b). To assess a dose–response effect, we compared NK cell levels after exposure to 0.5 Gy or 4 Gy γ-rays and observed a significant reduction in NK cell retention at 4 Gy relative to 0.5 Gy and control groups (Fig. S2c, d). Consistently, similar reductions in LP-resident NK cells were seen when comparing 4 Gy γ-rays with 0.5 Gy ^28^Si exposure (Fig. S2e). We further characterized maturation stages of NK cell subsets based on CD11b and CD27 expression (Fig. S1h, i). Marginal changes were observed after irradiation in CD11b^low^/CD27^high^ NK cell subset (Fig. S3a), whereas a significant increase was observed in CD11b^high^/CD27^low^ NK cell subset particularly after high-LET irradiation relative to control (Fig. S3b). In particular, the CD11b^high^/CD27^high^ NK cell subset was significantly decreased in the high-LET group compared to the γ-irradiated and control groups (Fig. 1h). Taken together, high-LET irradiation selectively and markedly reduced LP-resident NK cells, while other myeloid populations remained largely unaffected. Immunostaining confirmed a radiation quality–dependent loss of NK cells in the LP, an effect that was tissue-specific and evident at both early and late time points. High-LET exposure also reshaped NK cell maturation dynamics, with reduced CD11b^high^/CD27^high^ and increased CD11b^high^/CD27^low^ subsets. These findings indicate that high-LET irradiation disrupts NK cell homeostasis within the intestinal microenvironment.

### High-LET irradiation alters matrix-associated proteins that regulate immune cell retention in the mouse intestine

**Fig. 2:**
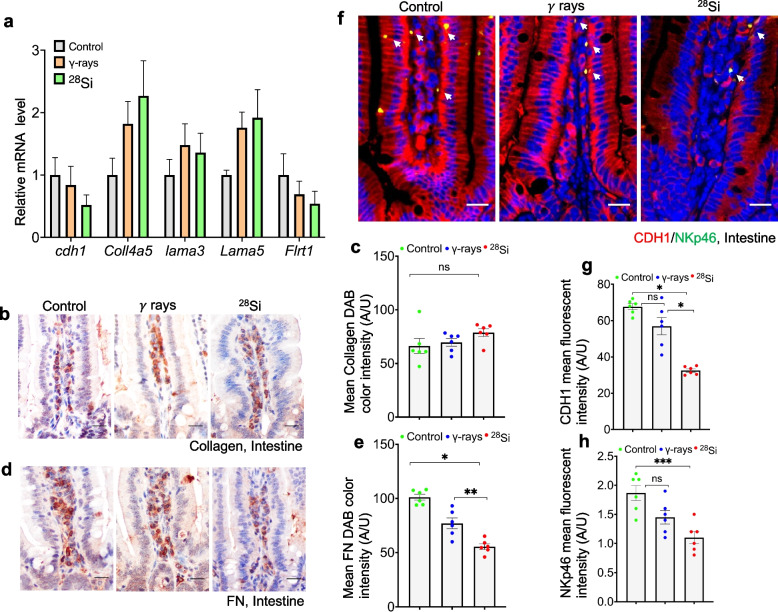
^28^Si-ion alters the matrix-associated proteins in mouse intestine 60 days after exposure. Detection of matrix-associated proteins at mRNA and protein levels in the mouse LP after irradiation (*n* = 5–6 per group). **a** Relative mRNA expression of *Cdh1*, *Collagen*, *Lama3, Lama5* and *Flrt1* at 60 days post-irradiation (*n* = 5). **b** Representative collagen immunostaining, and **c** quantification of mean color intensity (error bar = ± SEM, *n* = 6). **d** DAB-stained FN in mouse intestine, and **e** corresponding mean pixel intensity (error bar = ± SEM, *n* = 6). **f** Fluorescent co-staining of NKp46 (green) and CDH1 (red) at 60 days post-irradiation; white arrows mark resident NK cells. Nuclei were stained with DAPI (blue). **g–h** Quantification of fluorescence mean pixel intensity of CDH1 (**g**) and NK cells (**h**). In bright-field images, nuclei were counterstained with hematoxylin. Scale bar, 20 μm. Statistical analysis was performed using one-way ANOVA with Tukey’s post hoc test. Data are mean ± SEM. *p < 0.001; **p = 0.002; ***p = 0.0009, ns = non-significant

The intestinal microenvironment profoundly influences immune-cell retention. Matrix-associated proteins in the intestine play a critical role in NK cell recruitment and retention, which are essential for their functional activity [25]. To investigate this, we analyzed the mRNA expression levels of *E-cadherin* (*Cdh1)*, *Collagen (Col4a5), Fibronectin Leucine Rich Transmembrane Protein 1 (Flrt1)* and *Laminin (Lama3, Lama5)*. While *Col4a5*, *Lama3*, and *Lama5* mRNA levels showed a slight increase following irradiation, the changes were not statistically significant (Fig. 2a). In contrast, trends toward decreased Cdh1 and Flrt1 mRNA levels were observed after high-LET irradiation compared to the control (Fig. 2a). To validate these findings at the protein level, we performed immunostaining of intestinal tissue sections for CDH1, Collagen, and Fibronectin (FN). A modest increase in Collagen expression was observed after irradiation; though, this difference was not statistically significant compared to the control (Fig. 2b, c). However, both CDH1 and FN protein expression were reduced following irradiation, with decreasing trends observed in both the low-LET and high-LET groups compared to controls (Fig. 2d–g). Taken together, these findings indicate that high-LET irradiation decreases CDH1 and FN expression in the mouse intestine, potentially compromising NK-cell retention in the LP region (Fig. 2h).

### High-LET radiation exposure disrupts NK-cell regulatory molecules in mice at two months post-irradiation

**Fig. 3:**
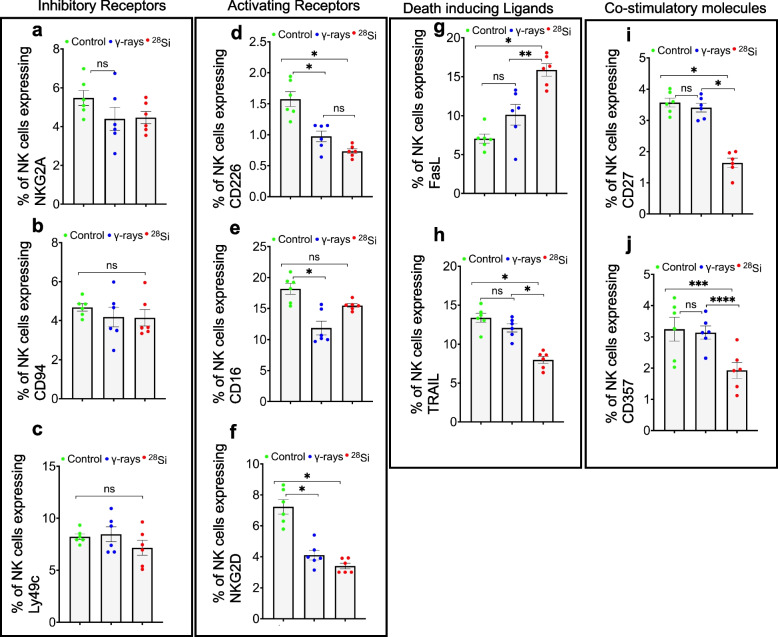
High-LET IR alter NK-cell receptors expression 60 days after exposure. Detection of cell-surface receptors on LP-resident NK cells was performed by flow cytometry (*n* = 6 per group). **a–c** Percentages of NK cells expressing the inhibitory receptors NKG2A, CD94 and Ly49c relative to controls. **d–f** Percentages of NK cells expressing the activating receptors CD226, CD16 and NKG2D. **g–h** Percentages of NK cells expressing the death-inducing ligands FasL and TRAIL compared to controls. **i–j**, Percentages of NK cells expressing the co-stimulatory molecules CD27 and CD357 after irradiation. Statistical analysis was performed using one-way ANOVA with Tukey’s post hoc test. Data are presented as mean ± SEM. *p < 0.001, **p = 0.0019, ***p = 0.0149, ****p = 0.025, ns = non-significant, *n* = 6

NK cell function is tightly regulated by a balance of inhibitory and activating receptors, death-inducing ligands, and co-stimulatory molecules [26, 27]. We analyzed the expression of key inhibitory receptors (NKG2A, CD94, and Ly49c), activating receptors (CD226, CD16, and NKG2D), death-inducing ligands (FasL and TRAIL), and co-stimulatory molecules (CD27 and CD357) in NK cells following irradiation. Inhibitory receptors (NKG2A, CD94, and Ly49c) showed minimal changes in expression after irradiation compared to control (Fig. 3a–c). In contrast, activating receptors (CD226, CD16, and NKG2D) were significantly downregulated following irradiation, with CD226 and NKG2D showing a radiation quality-dependent decrease in expression (Fig. 3d–f). Among the death-inducing ligands, FasL expression was upregulated, whereas TRAIL was downregulated specifically after high-LET irradiation (Fig. 3g, h). Co-stimulatory molecules CD27 and CD357 were also significantly downregulated in NK cells following high-LET irradiation, but remained largely unchanged after low-LET exposure (Fig. 3i, j). These findings suggest that high-LET irradiation disrupts the expression of key activating receptors, co-stimulatory molecules, and death-inducing ligands in NK cells, potentially impairing their ability to eliminate senescent or damaged cells.

### High-LET radiation attenuates NK-cell ligands in intestinal epithelial cells two months after exposure

**Fig. 4:**
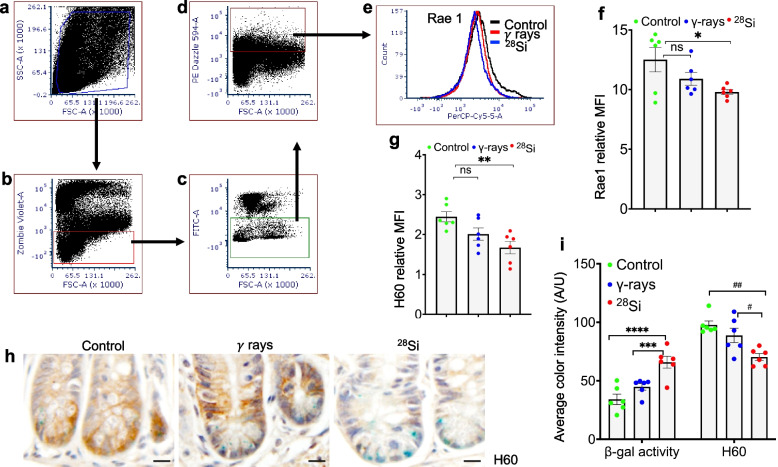
High-LET irradiation alters ligands for NK cell-activating receptors in IECs. Immunostaining of cell-surface ligands on IECs after irradiation, analyzed by flow cytometry and microscopy. **a–d**, Gating strategy for flow cytometry: **a** debris exclusion; **b** live-cell selection; **c** exclusion of CD45⁺ immune cells; and **d** identification of CDH1⁺ IECs. **e** Overlaid histograms showing the shift in Rae1 expression after irradiation. **f–g**, Mean fluorescence intensity of Rae1 (**f**) and H60 (**g**) expression in IECs. Senescent cells were identified using SA-β-galactosidase staining with concurrent detection of H60. **h** Representative bright-field images showing co-staining of H60 (brown) and SA-β-galactosidase (blue). **i** Quantification of DAB (H60) and β-galactosidase staining intensities. Nuclei were counterstained with hematoxylin. Scale bar, 20 μm. Statistical analysis was performed using one-way ANOVA with Tukey’s post hoc test. Data are mean ± SEM. *p = 0.038; **p = 0.0055; ***p = 0.008; ****p = 0.0002; ^#^p = 0.0235; ^##^*p* = 0.0013; ns = non-significant, *n* = 6

**Fig. 5:**
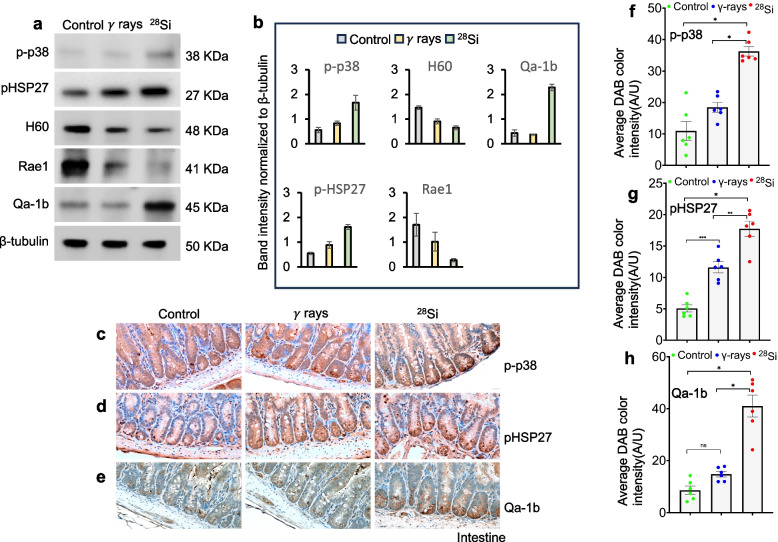
High-LET irradiation induces p38 MAPK signaling and downstream pathways in mouse intestine. Immunoblot and in situ detection of p38 MAPK and downstream signaling proteins in IECs after irradiation **a** Immunoblot analysis of phospho-p38, phospho-HSP27, H60, Rae1 and Qa-1b; β-tubulin served as a loading control. **b** Quantification of band intensities for phospho-p38, phospho-HSP27, H60, Rae1 and Qa-1b, normalized to β-tubulin (n = 5). **c–e** Immunohistochemical detection of phospho-p38 (**c**), phospho-pHSP27 (**d**), and Qa-1b (**e**). **f–h**, Average DAB staining intensities for phospho-p38 (**f**), phospho-HSP27 (**g**), and Qa-1b (**h**) presented as bar graphs. Scale bar, 20 μm. Statistical analysis was performed using one-way ANOVA with Tukey’s post hoc test. Data are presented as mean ± SEM. *p < 0.0001; **p < 0.0007, ***p < 0.0004, *n* = 6

NK cell function is regulated by activating ligands such as H60 and Rae1, which are expressed on target cells, including IECs [28]. We assessed H60 and Rae1 expression in irradiated and non-irradiated samples by flow cytometry and immunohistochemistry. Flow cytometry analysis revealed a moderate decrease in Rae1 expression and a significant reduction in H60 expression in IECs following high-LET irradiation (Fig. 4a–g). To further validate these findings, we performed co-staining for H60 in β-galactosidase (β-gal)-stained intestinal tissue sections. As previously reported, high-LET irradiation induced greater β-gal positivity in intestinal crypt epithelial cells compared to γ-irradiated and control groups, indicating increased senescence. Notably, H60 expression was significantly reduced in the crypt base following high-LET irradiation (Fig. 4h, i). Immunoblot analysis showed a similar decreasing trend for both Rae1 and H60 expression (Fig. 5a, b). These findings suggest that high-LET irradiation induces robust cellular senescence while concurrently downregulating H60 expression in senescent intestinal epithelial cells (IECs), a shift that potentially impairs NK cell-mediated recognition and clearance. Collectively, these data reveal a distinct molecular signature characterized by reduced H60 and Rae1 expression alongside an increased senescent burden following high-LET exposure.

### High-LET irradiation activates p38 MAPK signaling and upregulates Qa-1b expression in intestinal epithelial cells

Radiation is known to activate p38 MAPK signaling as part of a stress-adaptive response. We previously reported the activation of p38 MAPK signaling in the mouse intestine following exposure to high-LET ^28^Si irradiation [6]. To further investigate this, we performed immunoblot analysis of phosphorylated p38 and its downstream target, phosphorylated HSP27, 60 days after ^28^Si exposure. The results confirmed sustained activation of the p38 MAPK pathway in the irradiated intestine (Fig. 5a, b). To validate these findings in situ, we conducted immunodetection of phosphorylated p38 and phosphorylated HSP27 in intestinal tissue sections. Both proteins showed significantly increased expression in ^28^Si-irradiated tissues compared to controls, indicating persistent activation of the pathway (Fig. 5c, d, f, g). Additionally, immunoblot analysis revealed significant upregulation of Qa-1b in the intestines of high-LET irradiated mice (Fig. 5a, b). This increase was further confirmed by immunostaining, which showed enhanced Qa-1b expression specifically in IECs (Fig. 5e, h). Overall, these findings indicate that high-LET irradiation with ^28^Si induces sustained activation of the p38 MAPK pathway and upregulates Qa-1b expression in the intestinal epithelium, potentially contributing to immune modulation within the irradiated tissue.

### IR-induced p38 MAPK signaling impairs NK cell cytotoxicity via Qa-1b upregulation in IECs

**Fig. 6:**
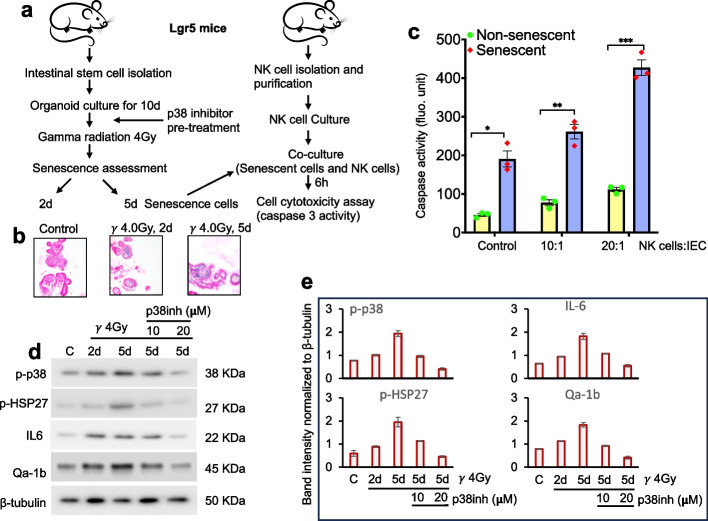
NK cells promote radiation-induced senescent IECs cytotoxicity in co-culture experiments. Optimization of radiation-induced senescence and assessment of in vitro NK-cell cytotoxicity. **a** Schematic illustration of IEC organoid culture, irradiation, senescence detection and NK-cell co-culture assays. **b** SA-β-galactosidase staining of IEC organoids at 2 and 5 days after 4 Gy γ-irradiation; nuclei were counterstained with Nuclear Red. **c** Caspase activity in NK cells co-cultured with senescent versus non-senescent IECs. The data presented are representative of three independent experiments. Statistical analysis was performed using one-way ANOVA with Tukey’s post hoc test. Error bars represent mean ± SEM. *p = 0.017; **p = 0.0046; ***p = 0.0023. **d** Immunoblot analysis of phospho-p38, phospho-HSP27, IL-6 and Qa-1b in IECs over a 0–5-day time course following 4 Gy γ-irradiation with or without p38 inhibitor treatment; β-tubulin served as a loading control. **e** Quantification of immunoblot band intensities, normalized to β-tubulin. Data presented are representative of three independent experiments

To investigate the mechanistic basis of IR-induced impairments in intestinal immune surveillance, we employed an intestinal organoid model irradiated with 4 Gy of γ-rays. This dose was selected based on our previous findings demonstrating that high-LET radiation is approximately 8- to 15-fold more effective than low-LET sources at inducing senescent cell accumulation in mouse intestine. Consequently, 4 Gy of γ radiation was used as biologically equivalent to 0.5 Gy of ^28^Si-ion [5, 6]. To validate this equivalency, we performed SA-β-gal staining in samples exposed to 0.5 Gy ^28^Si or 4 Gy γ-rays. As expected, SA-β-gal activity was significantly increased in the ^28^Si group compared to controls, consistent with earlier observations (Fig. S4a, b). Although SA-β-gal staining was slightly higher in the ^28^Si group than in the 4 Gy γ-ray group, the difference was not statistically significant (Fig. S4b). To further confirm these findings, we assessed Lamin B1 expression and observed a significant reduction in irradiated samples relative to controls; while no significant differences were detected between the two radiation groups (Fig. S4c, d). First, we assessed IR-induced senescence in cultured IECs at 2- and 5-days post-irradiation. Maximum senescence, as measured by β-galactosidase staining, was observed at 5 days post-IR in organoid cultures (Fig. 6a, b). We further validated radiation-induced senescence using p16 (CDKN2A) and Lamin B1 staining. The results showed that senescence peaked at 5 days post-irradiation, as indicated by elevated p16 expression and a corresponding reduction in Lamin B1 levels (Fig. S5a, b). Following induction of senescence, we co-cultured senescent and non-senescent IECs with freshly isolated allogeneic NK cells. Apoptosis was assessed by measuring active caspase-3. The data showed significantly higher caspase-3 activity in senescent IECs compared to non-senescent controls, particularly at an Effector (NK cells)-to-Target (Senescent cells) (E:T) ratio of 20:1, indicating enhanced NK cell-mediated killing of senescent cells (Fig. 6c).

**Fig. 7:**
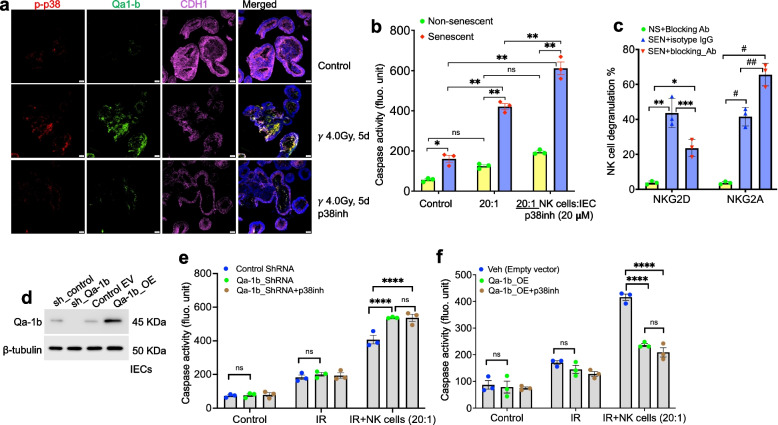
In vitro assessment of p38 MAPK inhibition as a potential approach to modulate NK-cell cytotoxicity. **a** Representative immunofluorescence images showing phospho-p38, IL-6 and CDH1 expression in IEC organoid sections 5 days after irradiation; nuclei were stained with DAPI. Scale bar, 20 μm. **b** Caspase activity in NK cells co-cultured with senescent or non-senescent IECs, with or without p38 inhibition. Data shown are representative of three independent experiments. Statistical analysis was performed using two-way ANOVA with Tukey’s post hoc test. (Error bars represent mean ± SEM.; *p = 0.0083; **p < 0.001; ns = not significant). **c** NK-cell degranulation (CD107a) in co-cultures with senescent or non-senescent IECs in the presence or absence of NKG2D- or NKG2A-blocking antibodies. Degranulation decreased with NKG2D blockade and increased with NKG2A blockade in the presence of senescent IECs. Data shown are representative of three independent experiments. Statistical analysis was performed using one-way ANOVA with Tukey’s post hoc test. (Error bars represent mean ± SEM.; *p = 0.0062; **p < 0.0001; ***p = 0.0054, ^#^*p* < 0.0001, ^##^*p* < 0.0013). **d** Immunoblot analysis of Qa-1b expression in transfected IECs. **e** Caspase activity in NK cells co-cultured with senescent or non-senescent IECs expressing Qa-1b shRNA or control ShRNA and treated with p38 inhibitor. Data shown are representative of three independent experiments. Statistical analysis was performed using two-way ANOVA with Tukey’s post hoc test. Error bars represent mean ± SEM.; ****p < 0.001; ns = not significant. **f** Caspase activity in NK cells co-cultured with senescent or non-senescent IECs stably expressing Qa-1b or empty vector and treated with p38 inhibitor. Data shown are representative of three independent experiments. Statistical analysis was performed using two-way ANOVA with Tukey’s post hoc test. (Error bars represent mean ± SEM.; ****p < 0.001)

To explore the role of p38 MAPK signaling in regulating this interaction, we pretreated intestinal organoids with or without a p38 MAPK inhibitor and followed them up to 5 days post-IR. Immunoblot analysis revealed a time-dependent increase in phosphorylated p38, phosphorylated HSP27, IL-6, and Qa-1b following IR (Fig. 6d, e). Treatment with the p38 inhibitor effectively suppressed p38 MAPK signaling and led to reduced expression of IL-6 and Qa-1b (Fig. 6d, e). The reduction in IL-6 suggests a decrease in the pro-inflammatory phenotype when p38 signaling is inhibited in irradiated organoids. Immunostaining of organoids further confirmed these findings, where IR induced expression of phospho-p38 and Qa-1b, along with a reduction in CDH1. Moreover, p38 inhibition blocked IR-induced phospho-p38 and Qa-1b expression (Fig. 7a). Notably, CDH1 levels remained unchanged in irradiated cells when p38 signaling was inhibited, suggesting that CDH1 downregulation occurs independently of the p38 MAPK pathway (Fig. 7a).

To determine whether suppression of Qa-1b via p38 inhibition enhances NK cell-mediated cytotoxicity, we repeated the co-culture experiments with senescent and non-senescent IECs pretreated with the p38 inhibitor. Caspase-3 activity was significantly increased in senescent IECs after p38 inhibition, suggesting enhanced NK cell killing, likely due to reduced Qa-1b expression (Fig. 7b). Further, we performed NK cell degranulation assays in the presence or absence of blocking antibodies. Pre-incubation of NK cells with an NKG2D-blocking antibody significantly reduced degranulation against senescent IECs compared to isotype control, confirming the importance of NKG2D-mediated activation. In contrast, blocking the inhibitory receptor NKG2A significantly enhanced NK cell degranulation in response to senescent IECs, suggesting that engagement of NKG2A by its ligand Qa-1b impairs NK cell function (Fig. 7c). Collectively, these findings demonstrate that IR-induced activation of p38 MAPK signaling promotes expression of Qa-1b in IECs, which suppresses NK cell cytotoxicity via NKG2A engagement. Inhibition of p38 signaling enhances NK cell-mediated clearance of senescent cells by downregulating Qa-1b, thereby restoring NKG2D-dependent immune surveillance (Fig. 7c).

To further investigate the underlying mechanism, we performed functional causality experiments by silencing or overexpressing Qa-1b in IEC for NK cell cytotoxicity assay. Immunoblot analysis confirmed efficient Qa-1b knockdown using shRNA and robust overexpression following transfection with Qa-1b (Fig. 7d). To test whether Qa-1b modulation alters NK cell–mediated cytotoxicity, we conducted co-culture assays using senescent and non-senescent IECs in which Qa-1b was either overexpressed or silenced, with or without p38 inhibitor. Caspase-3 activity was significantly increased in senescent IECs following Qa-1b knockdown, indicating enhanced NK cell–mediated killing, likely due to reduced Qa-1b expression (Fig. 7e). In contrast, Qa-1b overexpression markedly suppressed caspase-3 activation. Notably, p38 inhibition did not significantly alter caspase-3 activity in senescent IECs regardless of Qa-1b overexpression or knockdown (Fig. 7e, f). Over all these results demonstrate that senescent IECs upregulate the inhibitory ligand Qa-1b through p38 MAPK activation, suppressing NK-cell cytotoxicity via NKG2A engagement. p38 inhibition reduces Qa-1b expression and restores NK-mediated killing. Qa-1b knockdown increases, whereas overexpression decreases NK-cell cytotoxicity, and p38 inhibition has no additional effect when Qa-1b levels are altered, indicating Qa-1b is the key downstream effector of p38. These findings show that p38-driven Qa-1b expression enables senescent IECs to evade NK-cell surveillance.

## Discussion

**Fig. 8:**
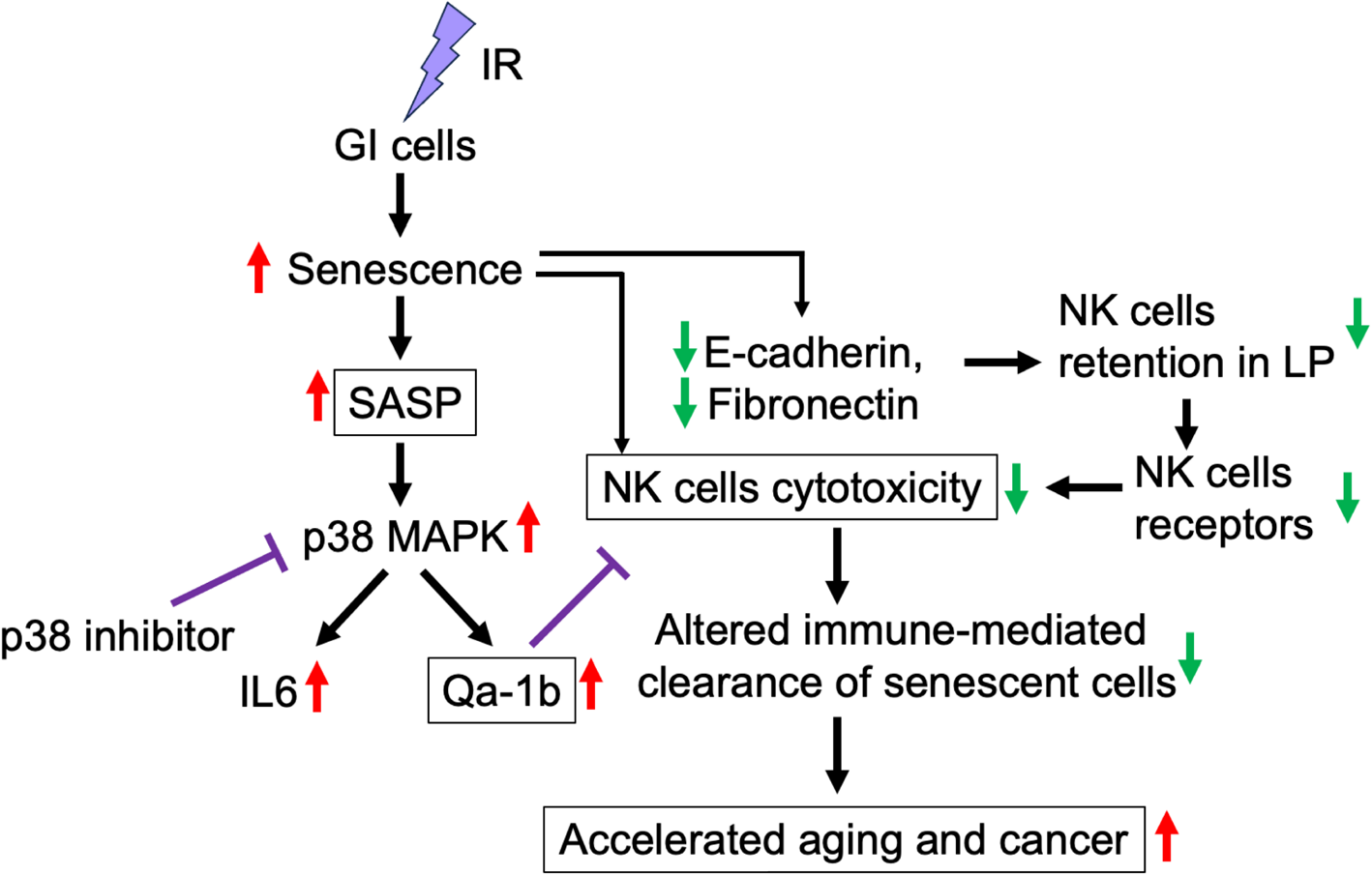
Schematic illustrations of the study findings. Red arrows indicate increased observations, while green arrows indicate decreased observations following high-LET irradiation. Purple blunt-ended line (T-bar) indicates inhibition or negative regulation. Note: IL-6 is part of the senescence-associated secretome rather than a direct upstream regulator of Qa-1b. Schematic presentation was generated using MS PowerPoint (Microsoft Corporation, CA USA)

Senescent cells commonly arise in response to injury or stress and are typically cleared by the immune system [29]. However, persistent senescence has been observed long after exposure to high-LET IR, suggesting impaired immune-mediated clearance [6]. This study is a continuation of our earlier work, in which we demonstrated long-term persistence of senescent GI stem cells using *Lgr5*-GFP reporter mice following irradiation [6]. Here, using the intestine as a model organ system, we investigate the mechanisms of immune evasion that may contribute to the sustained presence of senescent epithelial cells after high-LET IR exposure. We found a significant reduction in NK cells following ^28^Si irradiation, while macrophages, dendritic cells, and monocytes in the LP showed minimal changes. Expression of matrix-associated proteins, including FN and CDH1, was decreased in the LP, along with downregulation of activating NK cell receptors NKG2D and CD226. In contrast, inhibitory receptors (NKG2A, CD94, and Ly49c) remained unchanged. The expression of TRAIL, CD27, and CD357 molecules important for NK cell function was also reduced. Subtype analysis revealed a marked decline in CD11b^high^CD27^high^ NK cells, which are critical for recognizing cells expressing NKG2D ligands. Concurrently, epithelial expression of the NKG2D ligands Rae1 and H60 was decreased, while expression of the inhibitory ligand Qa-1b (which binds to NKG2A) was upregulated. Notably, Qa-1b upregulation correlated with increased p38 MAPK activation. Inhibition of p38 MAPK suppressed Qa-1b expression and restored NK cell cytotoxicity in vitro (Fig. 8).

These findings suggest that high-LET irradiation significantly impairs the immune surveillance within the LP, primarily by negatively impacting NK cell numbers and function. The most direct and concerning finding is the significant reduction in NKp46⁺ NK cells. NKp46 is an activating receptor crucial for NK cell recognition of target cell (senescent cells) [30]. A decrease in these cells directly translates to a diminished overall NK cell presence and potentially reduced ability to mount an effective immune response in the LP. Although NK cells exhibit the most pronounced functional impairment after high-LET radiation, it is possible that other immune cell populations, despite showing no significant quantitative decline, such as macrophages, may also experience functional alterations [31]. Moreover, the dynamics of crosstalk between NK and other immune and non-immune cells may also shift with a given decline in NK cell population [32–34]. The reduction in FN and CDH1 in the LP indicates potential damage to the structural integrity of the extracellular matrix, which could further hinder immune surveillance by affecting cell–cell interactions, immune cell trafficking, and tissue homeostasis [35–37].

Downregulation of activating receptors NKG2D and CD226 reduces the ability of remaining NK cells to recognize and kill target cells, while unchanged inhibitory receptors shift signaling toward an inhibitory state, making NK cells less responsive [38]. The decreased expression of TRAIL, CD27, and CD357 further compromises NK cell function, including cytotoxicity, maturation, and co-stimulation [39, 40]. The marked decline in CD11b^high^CD27^high^ NK cells signify a targeted impairment of a key NK cell population involved in anti-tumor and anti-viral immunity [41, 42]. We further show that epithelial cells actively reinforce this immunosuppressive state. Decreased epithelial NKG2D ligands (Rae1, H60) and upregulated Qa-1b is a highly significant and detrimental change in the epithelial microenvironment. Rae1 and H60 activating ligands that bind to NKG2D on NK cells, signaling for NK cell activation and killing [43, 44]. Their reduction means there are fewer "danger signals" for NK cells to recognize, further dampening NK cell activity. Qa-1b is an inhibitory ligand that binds to NKG2A on NK cells, leading to NK cell inhibition [45, 46]. Its upregulation provides a strong inhibitory signal to NK cells, effectively "turning them off" and preventing them from attacking healthy or transformed epithelial cells. This shift in activating and inhibitory ligands does not lead to indiscriminate killing. Instead, our data demonstrate that altered ligand ratios, rather than absolute expression levels, govern NK cell recognition and clearance of senescent IECs.

Mechanistic studies demonstrated direct correlation of Qa-1b upregulation with increased p38 MAPK activation which establishes a molecular pathway linking high-LET irradiation to the observed immune dysregulation [47, 48]. Our findings show that senescent IECs trigger activation of the p38 MAPK pathway, which leads to sustained expression of the inhibitory ligand Qa-1b and redirects NK-cell interactions toward NKG2A-dependent suppression. Mechanistically, Qa-1b emerges as the dominant downstream effector of p38: its knockdown restores NK-cell cytotoxicity, whereas overexpression dampens it, and p38 inhibition provides no further benefit once Qa-1b levels are experimentally altered. Together, our results support a model in which high-LET radiation establishes an immune-privileged senescent IECs niche through dual mechanisms: depletion and functional dysregulation of NK cells, coupled with epithelial reprogramming toward inhibitory signaling. This integrated response enables senescent cells to evade clearance and persist within the tissue microenvironment, with potential consequences for aging, tumorigenesis, and tissue dysfunction (Fig. 8).

From a translational perspective, these findings indicate that high-LET radiation concurrently impacts NK cell function and intestinal tissue homeostasis. In clinical settings, particularly carbon (^12^C)-ion radiotherapy, these results may highlight the challenge of preserving anti-tumor immunity while minimizing radiation-induced normal gastrointestinal tissue toxicity. Even typical low-LET radiation, such as used in abdominal radiotherapy, can trigger similar responses. The discovery that high-LET radiation impairs NK cells via senescent IECs through the p38-Qa-1b pathway suggests that targeted clearance of senescent IECs could alleviate Qa-1b-dependent NK suppression and preserve gastrointestinal immune surveillance. These observations support a combination approach in which p38 MAPK inhibitors and senolytic agents could be leveraged to achieve synergistic mitigation of radiotherapy-induced normal GI-tissue toxicity while maintaining anti-tumor immunity. More broadly, this study has implications for gut health in other pro-inflammatory disease conditions where senescent IECs accumulate.

Findings from this study should be interpreted within the context of several considerations and evolving understanding on NK cell-mediated immunosurveillance in the gut. The use of a single late time point and a single high-LET radiation condition limits insight into temporal dynamics and exposure heterogeneity. Mechanistic inferences regarding the p38-Qa-1b axis are derived primarily from in vitro and ex vivo systems and would benefit from further validation in in vivo settings. While the present study focuses on NK cells, contributions from additional immune and systemic factors remain to be fully elucidated. Moreover, analysis was confined to NK cell number and function within the lamina propria of intestinal mucosa, and examination of other tissue compartments may yield complementary insights. Finally, in light of emerging evidence for immunological divergence in murine Qa-1b function [49], further studies will be important to determine the extent to which these mechanisms are conserved in human tissues.

In conclusion, our study demonstrates that high-LET ^28^Si irradiation significantly impairs immune integrity in the intestinal LP by reducing NK cell numbers and function, marked by diminished activating receptor expression and upregulation of the inhibitory ligand Qa-1b through p38 MAPK activation. These findings highlight p38 MAPK as a potential therapeutic target, offering key insights for developing countermeasures in spaceflight and enhancing the efficacy of cancer radiotherapy.

## Materials and methods

### Animals experiment and radiation

Male *Lgr5*-EGFP-IRES-CreERT2 mice (Stock No. 008875) were obtained from The Jackson Laboratory (Bar Harbor, ME, USA) and housed at the Georgetown University (GU) Animal Care Facility as described earlier [6]. For this study, groups of *Lgr5*-EGFP-IRES-CreERT2 mice were subjected to whole-body irradiation with ^28^Si ions (energy: 300 MeV/nucleon; LET: 69 keV/μm; dose: 0.50 Gy) at the NASA Space Radiation Laboratory (NSRL), Brookhaven National Laboratory (BNL). Precise dose delivery was achieved through beam calibration and dosimetry performed by NSRL physicists, with exposure variability kept within ± 2.5%. For additional information on the procedures for beam calibration and dosimetry, please refer to https://www.bnl.gov/nsrl/userguide/dosimetry-calibration.php (accessed 15 August 2024). Additional groups received whole-body γ irradiation (0–4.0 Gy) using a ^137^Cs source, while control mice were sham-irradiated. All mice were transported to the BNL Animal Facility one week prior to irradiation to allow for acclimatization and were returned to the GU Animal Facility the day following radiation exposure. Transportation was carried out in a temperature-controlled environment to maintain physiological stability and ensure animal welfare. At both facilities, animals were maintained under standardized conditions, including temperature-controlled rooms set at 22 °C, 50% humidity, and a 12-h light/dark cycle, with ad libitum access to irradiated food and filtered water. All animal procedures were approved by the Institutional Animal Care and Use Committees of BNL (Protocol #345, approved October 12, 2021) and GU (Protocol #2016–1129, approved August 1, 2021). All experiments were conducted in accordance with the *Guide for the Care and Use of Laboratory Animals* (National Research Council, U.S. National Academy of Sciences).

### IECs, LP cells isolation and Flow-cytometry analysis

Mice were euthanized and small intestines were incised and IECs were isolated as described earlier [6]. Briefly, tissues were incubated in 2 mM EDTA in PBS for 20 min, followed by two vigorous washes with PBS. The supernatant containing villus cells was discarded. Tissues were then treated with a dissociation solution consisting of 1 mg/mL STEMxyme® 2 Collagenase/Neutral Protease (Dispase) (Cat# LS004112, Worthington Biochemical Corporation, Lakewood, NJ) and 0.1 mg/mL DNase I (Cat# 89,836, Thermo Scientific, Waltham, MA) in HBSS (Cat# 14175103, Thermo Scientific), and incubated at 37 °C for 20 min. After removing the supernatant, cells were released by vigorous shaking in cold HBSS. The resulting cell suspension was filtered through a 70 μm strainer, centrifuged, and washed twice with cold HBSS before being resuspended in HBSS supplemented with 2% FBS for organoid culture. LP immune cells were isolated as described earlier [50]. The LP cells were then enriched with a percoll gradient. For myeloid cell staining, dead cells were excluded using SYTOX™ Blue (cat#S34857, Life Technologies) and non-specific binding was blocked with Fc block anti-mouse CD16/32 Antibody (Clone 93, BioLegend). Samples were then surface stained at 4 °C for 30 min with the antibodies. Surface markers CD45, CD11c, NKp46, CD27, CD11b, CD64, NKG2A, CD94, Ly49c, Cd226, CD16, NKG2D, FasL, TRAIL CD357, CD11b, CDH1, H60 and Rae1 were used for flowcytometry analysis. Antibody details are mentioned in Supplementary Table S1. Cells were then washed with ice-cold PBS (supplemented with 10% FCS and 2 mM EDTA) at 400 g for 10 min at 4 °C and acquired immediately on BD Fortessa flow cytometer running FACS-Diva software (BD Bioscience). Analysis was performed using FCS express software version 7 (De Novo Software, Pasadena, CA). Average percentage of specific immune phenotypes was calculated from at least 6 mice per group.

### Immunohistochemistry and immunofluorescence

Paraffin-embedded intestinal tissue sections were first deparaffinized to remove embedding media, followed by rehydration through a graded series of ethanol solutions to restore tissue hydration. Antigen retrieval was then performed by heating the sections to boiling in citrate buffer (pH 6.0; Cat# 64,142–08, Electron Microscopy Sciences, Hatfield, PA, USA), a step that helps unmask epitopes and enhance antibody binding during subsequent staining procedures. Sections were then incubated overnight at 4 °C with primary antibodies specific for H60, phosphorylated p38 (p-p38), phosphorylated HSP27 (p-HSP27), and Qa-1b. Detection of antigen–antibody complexes was performed using the Mouse and Rabbit Specific HRP/DAB IHC Detection Kit (Cat# AB236466, Abcam), following the manufacturer's instructions. Following staining, tissue sections were counterstained with hematoxylin to visualize nuclei, then sequentially dehydrated through graded alcohols and cleared prior to mounting. Coverslips were applied using Permount mounting medium (Cat# SP15-100, Fisher Chemical) to preserve the samples and enhance optical clarity. Brightfield images were subsequently acquired using an Olympus microscope under appropriate imaging settings. For immunofluorescence analysis, tissue sections were incubated overnight at 4 °C with the primary antibodies, followed by washing and incubation with species-specific secondary antibodies conjugated to Alexa Fluor dyes: 488 (green), 594 (red), or 647 (far-red) for 1 h at room temperature. Nuclei were counterstained with DAPI to enable fluorescent visualization of nuclear structures. Slides were then examined and imaged using an Olympus fluorescence microscope under appropriate excitation and emission settings.

### IEC organoid culture, irradiation and cytotoxicity assays

Crypt cells were isolated and cultured in IntestiCult™ Organoid Media (Stem Cell Technologies, Catalog #06005) according to the manufacturer's instructions. Ten-day-old organoids were irradiated with 4.0 Gy γ rays, and cellular senescence was assessed at 2- and 5-days post-IR. For expression analysis and co-culture experiments, cells were treated with 0–20 µM SB 203580 (Thermo Fisher Scientific cat# 55–939-51MG) a selective p38 inhibitor. Non-irradiated cells were used as non-senescent controls, whereas senescent cells were collected 5 days after irradiation. Co-culture experiments were performed using Effector (NK cells)-to-Target (Senescent cells or non-senescent cells) at two ratios [(E:T); 10:1 and 20:1] as described earlier [48]. Cytotoxicity in target cells was assessed after 6 h of co-incubation by quantifying active caspase-3 activity using a spectrophotometer (EnzChek™ Caspase-3 Assay Kit; Cat# E13183, Thermo Fisher Scientific). Organoids incubated with medium only served as a negative control for baseline caspase-3 activity. Organoids treated with doxorubicin (1 µg/mL; Fisher Scientific) were used as positive controls. Alternatively, CD107a (lysosomal-associated membrane protein-1, LAMP-1) expression was used as a marker of NK cell degranulation, as described previously [48]. The degranulation data are presented as an index calculated as: (Sample Degranulation − Spontaneous Degranulation)/(Maximum Degranulation − Spontaneous Degranulation) × 100.

### Qa1-b overexpression and shRNA mediated knockdown

Coding sequence of mouse Qa-1b protein was synthesized and were inserted into mammalian expression plasmid (pcDNA3.1(+)-C-DYK) at *HindIII/ApaI* multiple cloning sites (Fig. S6) by GenScript, USA. The Qa-1 shRNA Plasmid (m) (sc-42923-SH) and Control shRNA Plasmid-A (sc-108060) procured from Santa Cruz Biotechnology. Mouse IECs were transfected with these constructs or control vectors (0.5 μg) using Effectene Transfection Reagent (Qiagen, 301,425) according to the manufacturer’s instructions. After 24 h, cultures were refreshed with new medium, and antibiotic selection was initiated after 48 h using puromycin (1.0 µg/mL; Santa Cruz, sc-108071) or neomycin (400 µg/mL; Santa Cruz, sc-255391). Following 5 days of selection, cells were returned to fresh growth medium, and Qa-1b overexpression or knockdown was confirmed by immunoblotting. IECs with Qa-1b knockdown or Qa-1b overexpression were further used for radiation exposure and cytotoxicity assay as described above.

### Imaging, quantification, and statistical analysis

Appropriate controls were included with all experimental samples to verify the specificity of immunostaining and to distinguish true signal from background staining. These controls included sections processed in parallel under identical conditions to ensure consistency and reliability of the staining procedure. For both immunohistochemistry and immunofluorescence, randomly selected regions of normal mucosa were imaged from each section using cellSens Entry v1.15 (Olympus Corp., Center Valley, PA, USA), as previously described [51, 52]. Multiple fields per section were captured to provide a representative assessment and minimize sampling bias. The number of technical and biological replicates is indicated in the figure legends. Statistical analyses were performed using Student’s *t*-test or one-way analysis of variance (ANOVA), or two-way ANOVA as appropriate, followed by Tukey’s post hoc test. All analyses were conducted using GraphPad Prism version 10.6.1 (GraphPad Software, Boston, MA, USA). All the experimental data are presented as mean ± standard error of the mean (SEM). A *p*-value of ≤ 0.05 used the determine the statistical significance between experimental groups.

## Supplementary Information

Supplementary Material 1.

## Data Availability

The research data generated or analyzed during this study are included in this published article and its Supplementary Material. Other data from the corresponding authors are available upon reasonable request.
