# Supplementary material for: High-linear energy transfer radiation disrupts natural killer cell surveillance of senescent intestinal cells in the mouse intestine
Source: Mol Biomed. 2026 Apr 7;7:45. doi: 10.1186/s43556-026-00446-z (PMC13057138; doi:10.1186/s43556-026-00446-z)
Supplement: Supplementary file 1 — Supplementary Material 1. [file 43556_2026_446_MOESM1_ESM.docx]

**High-Linear Energy Transfer radiation disrupts Natural killer cell surveillance of senescent intestinal cells in the mouse intestine**

Santosh Kumar^1*^, Shubhankar Suman^1^, Heng-Hong Li^1^, Jerry Angdisen^1^, Kamal Datta^1^, Albert J. Fornace Jr.^1,2^

^1^Department of Oncology, Lombardi Comprehensive Cancer Center, Georgetown University Medical Center, Washington, DC 20057, USA. ^2^Department of Biochemistry and Molecular & Cellular Biology, Georgetown University Medical Center, Washington, DC 20057, USA.

**Key Words:** NK cells, High-LET radiation, senescence, Lamina propria, p38 MAPK signaling, activating receptors, ligand Qa-1b

***Correspondence:**

Santosh Kumar, Ph.D., Assistant Professor, Department of Oncology, Lombardi Comprehensive Cancer Center, Georgetown University Medical Center, Research Building, Room E504, 3970 Reservoir Rd., NW, Washington, DC 20057, USA. Email: [sk1426@georgetown.edu](mailto:sk1426@georgetown.edu) Phone: +1-202-687-5216, Fax: 202-687-3140

**Supplementary Figure**

Figure S1

**Figure S1:** Gating strategy implemented to identify various immune cell types in the lamina Propria (LP). (A) Exclusion of debris, (B) Selection of live cells, (C) Selection of CD45-expressing immune cells, (D) Identification CD45^+^/CD64^+^ monocytes, (E) Identification CD45^+^/NKp46^+^ NK cells, (F) Identification CD45^+^/CD11c^+^ dendritic cells, (G) Identification CD45^+^/F4/80^+^ macrophages, (H) Analyzing NK cell subtypes using CD11b, CD27 markers. (I) Representation of multiple NK cell maturation stages based on CD11b and CD27 expression.

Figure S2

**Fig. S2** Assessment of NK-cell retention in the LP at 5 and 60 days after irradiation. **a–b** Representative bright-field images of mouse intestine showing time dependent NK-cell retention in the LP; nuclei were counterstained with hematoxylin. Scale bar, 20 μm. **c–e**, Representative bright-field images of mouse intestine showing dose dependent NK-cell retention in the LP; nuclei were counterstained with hematoxylin. Quantification of NK cells, analyzed by one-way ANOVA with Tukey’s post hoc test. Data are presented as mean ± SEM. Statistical significance: ******p* = 0.0004, *******p* = 0.0011, ********p* < 0.0001, *********p* = 0.0002,

n = 6.

Figure S3

**Fig S3:** Analysis of NK cell subtypes in the mouse LP 60 days after high-LET irradiation. (A) Bar graph showing the percentage change in the CD11b^low^/CD27^high^ population relative to control group. (B) Bar graph showing the percentage change in the CD11b^high^/CD27^low^ population relative to control group. Quantification of NK cells, analyzed by one-way ANOVA with Tukey’s post hoc test. Data are presented as mean ± SEM. Statistical significance: ******p* = 0.0176, *******p* = 0.0426, ********p* = 0.0055, ns = not significant, n = 6.

Figure S4

**Fig. S4** Assessment of senescence in intestinal cells to determine dose equivalency after irradiation. **a** SA-β-galactosidase staining in intestinal sections 60 days after exposure to ^28^Si (0.5 Gy) or γ-rays (4.0 Gy). **b** Mean pixel intensity of SA-β-galactosidase staining represented as a bar graph. **c** Representative images of Lamin B1 staining in mouse intestine after irradiation. **d** Mean fluorescence intensity of Lamin B1 staining represented as a bar graph. n = 6 per group. Statistical analysis was performed using one-way ANOVA with Tukey’s post hoc test. Data are presented as mean ± SEM. **p* < 0.0001, ns = not significant, n = 6.

Figure S5

**Fig. S5.** Assessment of radiation-induced senescence in IEC organoids using Lamin B1 and p16 staining. **a** Representative image showing p16 expression in IECs within cultured organoids. The data demonstrate increased p16 expression at 5 days after irradiation. **b** Representative images showing Lamin B1 expression in IEC organoids 5 days after irradiation. The data reveal a reduction in Lamin B1 expression following irradiation, consistent with enhanced senescence.

Figure S6

**Fig. S6** Schematic representation of the full-length Qa-1b coding sequence cloned into the mammalian expression vector pcDNA3.1(+)-C-DYK at the *HindIII*/*ApaI* multiple cloning sites. The vector map was generated using NGS data with SnapGene® software (Dotmatics; available at snapgene.com).

**Additional materials and method**

**Immunoblot analysis**

Protein lysates were prepared from isolated or cultured IECs using RIPA buffer containing protease and phosphatase inhibitor cocktail. Equal amounts of protein were separated by SDS-PAGE and transferred to PVDF membranes as previously described [1]. For immunoblot analysis, primary antibodies included anti-phospho-p38, anti-phospho-HSP27, anti-H60, anti-Rae1, anti-Qa-1b, anti-IL-6, and anti-β-tubulin (all from Santa Cruz Biotechnology). Secondary antibodies, including HRP-conjugated anti-rabbit, anti-mouse, and anti-goat IgG (all from Santa Cruz Biotechnology), were used. Immunoblots were developed using an enhanced chemiluminescence (ECL) detection system (Cat# 34080, Thermo Fisher Scientific). Signals were captured on an Amersham Imager (GE Life Sciences) and densitometrically analyzed using ImageJ2 software fiji version: 2.14.0/1.54f as described earlier [2].

**RNA isolation and qPCR analysis**

Total RNA was extracted from isolated IECs using RNeasy mini kit (Cat# 74104, Qiagen, Germantown, MD) and was reverse transcribed to cDNA using RT2 First Strand Kit (Cat# 330421; Qiagen, Valencia, CA) as described earlier [1]. qPCR analysis of matrix associated genes particularly *Cdh1, Collagen* and isoforms of *Laminin* was performed using specific primers (Supplementary Table S2) and SYBR green master mix (Qiagen) on CFX96 real-time instrument (Bio-Rad, Hercules, CA) using the temperature settings: 95 °C for 5 min and then 40 cycles of 95 °C for 15 s and 58 °C for 1 min. We employed the ΔΔCt method, in which ΔCt was calculated using the *Gapdh* reference gene and ΔΔCt was calculated relative to the control group as described earlier [1]. Changes in gene expression data are presented as fold change relative to control ± standard error of the mean (SEM).

**Senescence-associated (SA) β-Galactosidase staining**

β-Galactosidase staining of intestinal tissue was performed as previously described with minor modifications [3, 4]. Briefly, the intestines were flushed with phosphate-buffered saline (PBS) to remove fecal content. One-inch segments of the jejunum were excised and immediately fixed for 2 hours at 4°C in an ice-cold fixative solution containing 1% formaldehyde, 0.2% glutaraldehyde, and 0.02% NP-40 in PBS, using a rolling platform. After fixation, tissues were washed twice with PBS for 20 minutes at room temperature (20°C) on a rolling platform. Following the washes, tissues were incubated overnight at room temperature in the dark with X-gal staining solution consisting of 5 mM potassium ferricyanide (K₃Fe(CN)₆), 5 mM potassium ferrocyanide trihydrate (K₄Fe(CN)₆·3H₂O), 2 mM MgCl₂, 0.02% NP-40, 0.1% sodium deoxycholate, and 1 mg/ml X-gal, prepared in PBS adjusted to pH 5.6. After staining, tissues were washed twice in PBS for 20 minutes at room temperature, then post-fixed overnight in 10% neutral buffered formalin at 4°C in the dark on a rocking platform. Stained tissues were transferred to tissue cassettes and embedded in paraffin using standard histological procedures. Sections of 5 µm thickness were cut, immune-stained for H60 and nuclei were counterstained with hematoxylin prior to microscopic analysis.

| **Catalog#**  Table S1: Antibodies and reagent list | **Antibodies** | **Source** | **applications** |
| --- | --- | --- | --- |
| 103125 | Pacific Blue™ anti-mouse CD45 Antibody | BioLegend | Flowcytometry |
| 137627 | Alexa Fluor® 647 anti-mouse CD335 (NKp46) Antibody | BioLegend | Flowcytometry |
| 137607 | APC anti-mouse CD335 (NKp46) Antibody | BioLegend | Flowcytometry |
| 137611 | Brilliant Violet 421™ anti-mouse CD335 (NKp46) Antibody | BioLegend | Flowcytometry |
| 156529 | Spark NIR™ 685 anti-mouse NK-1.1 Antibody | BioLegend | Flowcytometry |
| 139319 | PE/Dazzle™ 594 anti-mouse CD64 (FcγRI) Antibody | BioLegend | Flowcytometry |
| 139307 | PerCP/Cy5.5 anti-mouse CD64 (FcγRI) Antibody | BioLegend | Flowcytometry |
| 117317 | PE/Cy7 anti-mouse CD11c Antibody | BioLegend | Flowcytometry |
| 100227 | Brilliant Violet 421™ anti-mouse CD3 Antibody | BioLegend | Flowcytometry |
| 123111 | PE/Cy5 anti-mouse F4/80 Antibody | BioLegend | Flowcytometry |
| 123119 | Alexa Fluor® 488 anti-mouse F4/80 Antibody | BioLegend | Flowcytometry |
| 147309 | PE/Cy7 anti-mouse/human CD324 (E-Cadherin) Antibody | BioLegend | Flowcytometry |
| 147301 | Alexa Fluor® 594 anti-mouse/human CD324 (E-Cadherin) Antibody | BioLegend | Flowcytometry |
| 130107 | PE anti-mouse RAE-1γ Antibody | BioLegend | Flowcytometry |
| 142803 | PE anti-mouse CD159a (NKG2AB6) Antibody | BioLegend | Flowcytometry |
| 105509 | PE/Cy7 anti-mouse CD94 Antibody | BioLegend | Flowcytometry |
| 108205 | FITC anti-mouse Ly-49C/F/I/H Antibody | BioLegend | Flowcytometry |
| 115711 | FITC anti-mouse CD314 (NKG2D) Antibody | BioLegend | Flowcytometry |
| 128809 | APC anti-mouse CD226 (DNAM-1) Antibody | BioLegend | Flowcytometry |
| 101327 | APC/Cy7 anti-mouse CD16/32 Antibody | BioLegend | Flowcytometry |
| 106805 | PE anti-mouse CD178.1 (FasL) Antibody | BioLegend | Flowcytometry |
| 109311 | PE/Cy7 anti-mouse CD253 (TRAIL) Antibody | BioLegend | Flowcytometry |
| 126311 | APC anti-mouse CD357 (GITR) Antibody | BioLegend | Flowcytometry |
| 124225 | APC/Cy7 anti-mouse/rat/human CD27 Antibody | BioLegend | Flowcytometry |
| 101225 | APC/Cy7 anti-mouse/human CD11b Antibody | BioLegend | Flowcytometry |
| 480049 | MojoSort™ Mouse NK Cell Isolation Kit | BioLegend | Flowcytometry |
| 158003 | PE anti-mouse CD16 Antibody | BioLegend | Flowcytometry |
| FAB1155P | Mouse H60 PE-conjugated Antibody, R&D Systems™ | Fisher Scientific | Flowcytometry |
| BDB566986 | CD107a (LAMP-1) Mouse, anti-Mouse, R718, Clone: 1D4B, BD Horizon™ | Fisher Scientific | Flowcytometry |
| 50-145-98 | CD314 (NKG2D) Rat anti-Mouse, Functional Grade, Clone: CX5, eBioscience | Fisher Scientific | neutralizing antibody |
| 50-146-15 | NKG2A/C/E Monoclonal Antibody (20d5), Functional Grade, eBioscience™ | Fisher Scientific | neutralizing antibody |
| 50-112-9472 | Rat IgG2a kappa Isotype Control (eBR2a), eBioscience™ | Fisher Scientific | neutralizing antibody |
| PIPA547081 | H60 Polyclonal Antibody, Invitrogen™ | Fisher Scientific | Immunohistochemistry |
| PIPA541738 | RAE1 Polyclonal Antibody, Invitrogen™ | Fisher Scientific | Immunohistochemistry |
| AF2225-SP | Mouse NKp46/NCR1 Antibody, R&D Systems™ | Fisher Scientific | Immunohistochemistry |
| BDB559829 | Qa-1(b) Mouse anti-Mouse, Biotin, Clone: 6A8.6F10.1A6, BD | Fisher Scientific | Immunoblot and Immunohistochemistry |
| PI21134 | Thermo Scientific™ Pierce™ High Sensitivity Streptavidin-HRP, Pre-Diluted | Fisher Scientific | Immunoblot |
| AP189PMI | IgG Donkey anti-Rat, HRP, Polyclonal, Secondary Antibody, MilliporeSigma™ | Fisher Scientific | Immunoblot |
| 55-939-51MG | MilliporeSigma™ Calbiochem™ SB 203580, Hydrochloride | Fisher Scientific | cell culture |
| A5019001 | Gibco™ CTS™ NK-Xpander™ Medium | Fisher Scientific | cell culture |
| D2893 | Invitrogen™ C12FDG (5-Dodecanoylaminofluorescein Di-β-D-Galactopyranoside) | Fisher Scientific | β-galactosidase assay |
| sc-9104 | β-tubulin | Santa Cruz Biotechnology Inc | Immunoblot |
| sc-166182 | p-p38 MAPK Antibody (E-1) | Santa Cruz Biotechnology Inc | Immunoblot |
| sc-166693 | p-HSP 27 Antibody (B-3) | Santa Cruz Biotechnology Inc | Immunoblot |
| sc-33709 | Laminin β-1 Antibody (LT3) | Santa Cruz Biotechnology Inc | Immunoblot |
| sc-8422 | Fibronectin Antibody (EP5) | Santa Cruz Biotechnology Inc | Immunoblot |
| sc-293182 | COL1A1 Antibody (3G3) | Santa Cruz Biotechnology Inc | Immunoblot |
| sc-2314 | donkey anti-mouse IgG-HRP | Santa Cruz Biotechnology Inc | Immunoblot |
| ab76055 | Anti-E Cadherin antibody [M168] (ab76055) | Abcam | Immunofluorescence |
| orb6210 | IL6 antibody | Biorbyt | Immunoblot |
| PA5-20379 | P16INK4a antibody | INVITROGEN | Immunoblot |
| 66095-1-1g | Lamin B1 Monoclonal antibody | Proteintech | Immunoblot |

Table S2: Primer details

| Catalog Number | Primer sequence | Source |
| --- | --- | --- |
| Item# VMPS-1007 | *Cdh1*: FOR 5' TCTTGGCGTTTCTTTCAAAC; REV 5' CAAAGATTCCAGCCAGAAAA | realtime primers.com |
| Item# VMPS-1286 | *Col4A5:* FOR 5' TGCTGACTAGCAACCATGAA, REV 5' TACTTCCTCAAACCGCACTC | realtime primers.com |
| Item# VMPS-3426 | *Lama3*: FOR 5' GGACCAGGTGATCTCTGATG; REV 5' CTGTCTCCTCCACAACTGCT | realtime primers.com |
| Item# VMPS-3427 | *Lama5*: FOR 5' GCTTTGAGAAGCAGTTCAGC; REV 5' CCAGAGCCGAAGTCATAGAA | realtime primers.com |
| *Item# VMPS-2217* | *Flrt1*: FOR 5’ TCAAAAGACCTCTGCCACTC; REV 5' CCGGGTCCATTTCTTTCTAT | realtime primers.com |
| *Gapdh* | *Gapdh* FOR 5’ GTGGAGTCATACTGGAACATGTAG; REV 5’ AATGGTGAAGGTCGGTGTG | Eurofins |

**References**

1. Kumar S, Suman S, Fornace AJ, Datta K. Space radiation triggers persistent stress response, increases senescent signaling, and decreases cell migration in mouse intestine. Proc Natl Acad Sci U S A. 2018;115(42):E9832–41. https://doi.org/10.1073/pnas.1807522115.
2. Kumar, S.; Kumar, K.; Angdisen, J.; Suman, S.; Kallakury, B.V.S.; Fornace, A.J., Jr. cGAS/STING Pathway Mediates Accelerated Intestinal Cell Senescence and SASP After GCR Exposure in Mice. Cells. 2025,14,1767. <https://doi.org/10.3390/cells14221767>
3. Kumar S, Suman S, Angdisen J, Moon BH, Kallakury BVS, Datta K, et al. Effects of High-Linear-Energy-Transfer Heavy Ion Radiation on Intestinal Stem Cells: Implications for Gut Health and Tumorigenesis. Cancers (Basel). 2024;16(19):3392.
4. Barker N, van Es JH, Kuipers J, Kujala P, van den Born M, Cozijnsen M, et al. Identification of stem cells in small intestine and colon by marker gene Lgr5. Nature. 2007;449(7165):1003–7. https://doi.org/10.1038/nature06196.
